# Absence of p300 induces cellular phenotypic changes characteristic of epithelial to mesenchyme transition

**DOI:** 10.1038/sj.bjc.6603202

**Published:** 2006-07-11

**Authors:** D Krubasik, N G Iyer, W R English, A A Ahmed, M Vias, C Roskelley, J D Brenton, C Caldas, G Murphy

**Correction to**: *British Journal of Cancer* (2006) **94**, 1326–1332. doi:10.1038/6603101

Owing to an author error, the incorrect Figure 5 was included in the paper. The correct version of Figure 5 is given below.

## Figures and Tables

**Figure 5:**
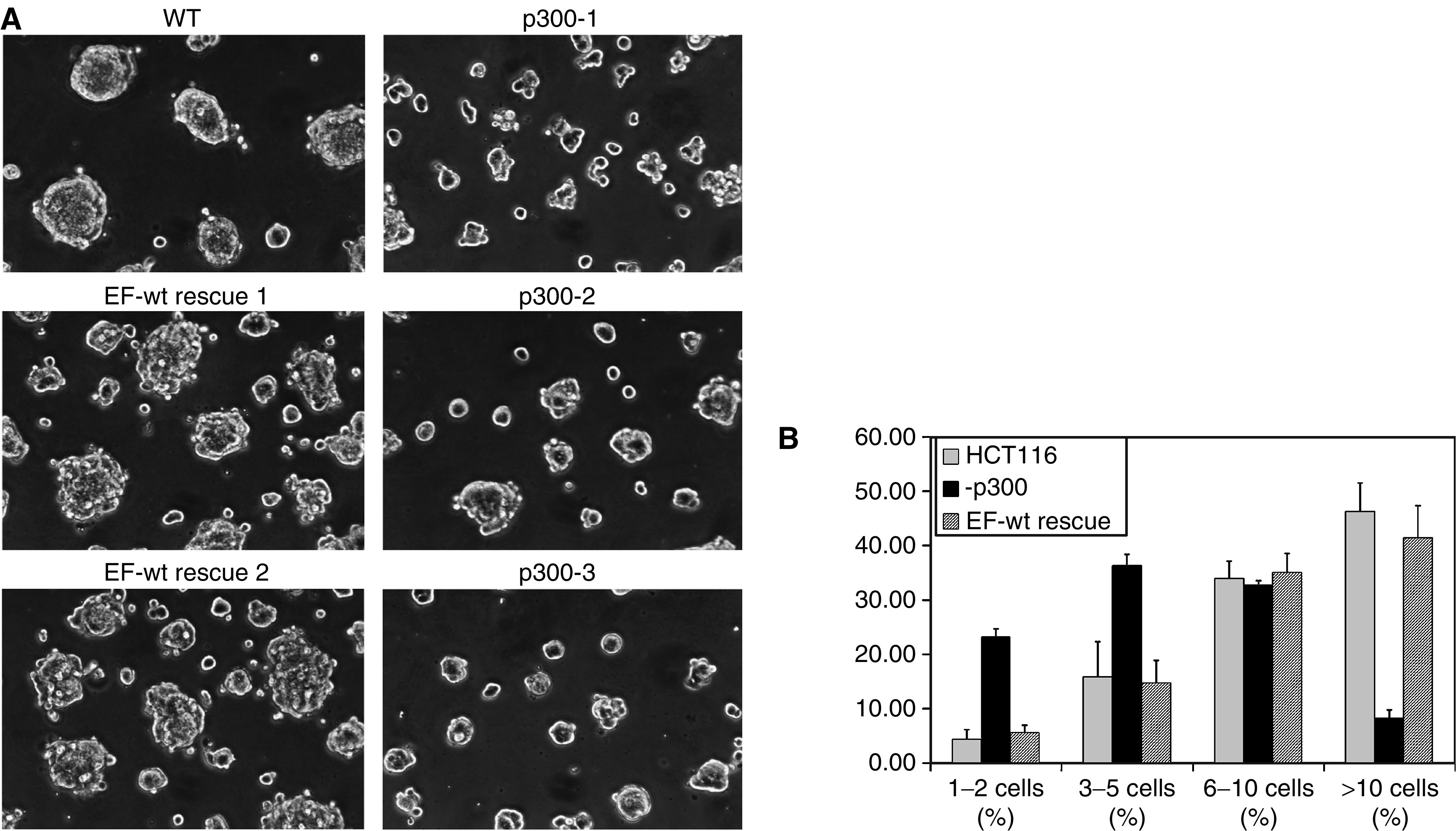
Adhesion defect on Matrigel growth after 18 h. (**A**) Matrigel growth of WT, p300^−^ and EF-wt rescue cell clusters. p300^−^ clones (p300-1, p300-2 and p300-3) cluster poorly into small clusters with ragged edges compared to WT cells. This defect is reversed in EF-wt rescue clones. Experiments were performed in triplicate, and the figure above shows a representative example. (**B**) Graph of cluster size distribution in HCT116, p300^−^ and EF-wt rescue cells. The graph demonstrates the distribution of cluster sizes in HCT116 *vs* the p300- and EF-wt rescue clones. Experiments were performed in triplicate in all three p300- clones (p300-1, p300-2 and p300-3) and two EF-wt rescue clones (EF-wt rescue-1 and EF-wt rescue-2). Error bars indicate one standard deviation.

